# “We can’t share things with our teachers”: Narratives of mistrust and disconnect between South African female learners and their teachers

**DOI:** 10.3389/feduc.2022.882959

**Published:** 2022-09-07

**Authors:** Zoe Duby, Kealeboga Maruping, Kim Jonas, Tracy McClinton Appollis, Lieve Vanleeuw, Catherine Mathews

**Affiliations:** 1 Health Systems Research Unit, South African Medical Research Council, Cape Town, South Africa; 2 Division of Social and Behavioural Sciences in the School of Public Health and Family Medicine, University of Cape Town, Cape Town, South Africa; 3 Adolescent Health Research Unit, Division of Child and Adolescent Psychiatry, University of Cape Town, Cape Town, South Africa; 4 Office of Aids and TB Research, South African Medical Research Council, Cape Town, South Africa

**Keywords:** adolescent girls and young women, teachers, South Africa, school connectedness, teacher connectedness, education, sexual and reproductive health (SRH)

## Abstract

The quality and nature of student-teacher relationships have implications outside of the academic domain. Support from teachers plays a significant protective role in the mental and emotional well-being of adolescents and young people, and can help to reduce or delay their engagement in risk behaviours, thereby decreasing negative sexual and reproductive health outcomes such as teenage pregnancy. Using the theory of teacher connectedness, an element of school connectedness, this research explores the narratives surrounding teacher-student relationships amongst South African adolescent girls and young women (AGYW) and teachers. Data were collected through in-depth interviews with 10 teachers, and 63 in-depth interviews and 24 focus group discussions with 237 AGYW aged 15–24 from five South African provinces characterised by high rates of HIV and teenage pregnancy amongst AGYW. Analysis of the data followed a thematic and collaborative approach, comprising coding, analytic memo-ing, and verification of emerging interpretations through discussion and participant feedback workshops. Findings related to perceptions of support and connectedness in teacher-student relationships centred around AGYW narratives of mistrust and a lack of support from teachers, and the consequential negative implications for academic performance and motivation to attend school, self-esteem, and mental health. Teachers’ narratives centred around challenges providing support, feeling overwhelmed and incapable of fulfilling multiple roles. Findings provide valuable insight into student-teacher relationships in South Africa, their impact on educational attainment, and on the mental health and sexual and reproductive health of AGYW.

## Introduction

Inconsistent school attendance, poor-quality learning, and low levels of educational attainment undermine health and wellbeing during adolescence, and throughout an individual’s life course. South Africa has joint epidemics of HIV and unintended teenage pregnancies; with a quarter of all new HIV infections occurring amongst adolescent girls and young women (AGYW) aged 15–24 (Duby et al., 2020). Education is a key structural driver of HIV amongst AGYW, and low levels of educational attainment are associated with higher risks of unintended pregnancies (The Global Fund, 2015). South Africa has high rates of teenage pregnancy, posing a threat to gender parity in education and resulting in poor mental health outcomes and low educational achievement amongst AGYW; figures suggest that a third of female learners drop out of school due to pregnancy (Bhana et al., 2010; Reddy et al., 2016; Jonas et al., 2020).

Social support theories outline the way in which individuals are nested within social support networks comprising of close bonds with people who provide support, a critical aspect of mental health and well-being (García-Moya, 2020). Close, positive, and supportive relationships with non-familial adults have been shown to serve a protective function for adolescents, enabling them to develop behavioural and social-emotional competencies (Baker et al., 2008; Herrero Romero et al., 2019; García-Moya, 2020). This is especially the case in situations where support from primary caregivers is insufficient or lacking (Herrero Romero et al., 2019). Teachers are in a prime strategic position to become significant non-familial adults in young people’s lives (García-Moya, 2020). There is increasing recognition that teachers, as well as providing academic instruction, are in a position to facilitate the social and emotional development of students (Binfet and Passmore, 2017).

As a theoretical framework, we focus on “teacher connectedness,” a sub-domain of “school connectedness.” School connectedness refers to the sense of belonging and connectedness within the school environment, encompassing the emotional bonds students have within the school setting, the extent to which they are respected and supported, and the perception that adults in the school environment care not only about their learning, but also about them as individuals (Rawatlal and Petersen, 2012; Biag, 2016; Mitchell et al., 2016; Sharp et al., 2019). Conversely, the theoretical construct of school connectedness can also help to explain the relationship between feelings of isolation, alienation and disconnect that some students may experience (Van Maele and Van Houtte, 2011; Page et al., 2021). Outside of the family context, the school environment has been shown to be important not only for academic development, but also to provide a protective environment for the social, psychological, and physical well-being of learners (Govender et al., 2013). Students’ sense of connectedness and engagement with the school environment is greatly influenced by their perceptions of interpersonal relationships with teachers, the strength of the bonds they have with teachers, and the extent to which they feel supported (Van Maele and Van Houtte, 2011; Mitchell et al., 2016).

Some theorists have emphasised the importance of delineating between school connectedness, relating to bonding and engagement in the school environment as a whole, and teacher connectedness, referring specifically to the sense of connectedness students have with their teachers (Garciìa-Moya et al., 2019). School connectedness has more to do with students’ overall feelings toward the educational institutional and school environment, whereas teacher connectedness is a dimension of the interpersonal relational domain (García-Moya, 2020).

In this paper we focus on teacher connectedness, referring to student-teacher bonds fostered by teachers through providing positive feedback, demonstrating empathy, and being open to dialogue (Sturdivant, 2020). Positive teacher-student relationships, characterised by feelings of trust and relatedness, are associated with positive academic motivation, successful expectations and self-belief, interest and satisfaction with school, and academic self-efficacy and performance (Gillespie, 2002; Baker et al., 2008; Mitchell et al., 2016). Student-teacher relationships have potentially significant and far-reaching implications outside of the academic domain; the support that teachers can provide is not only educational or academic, but can also be psychosocial or emotional support, playing a significant protective role in mental and emotional well-being, positively affecting students’ self-confidence, self-esteem, social skills, and social competence (Gillespie, 2005; Biag, 2016; Binfet and Passmore, 2017; Herrero Romero et al., 2019; Ungar and Theron, 2019; Kincade et al., 2020). Self-esteem, well-being, and perceived social support are key to ensure positive sexual and reproductive health (SRH) outcomes for adolescent girls and young women (Duby et al., 2021). Support from teachers can help to prevent or delay adolescents’ engagement in various high risk health behaviours (McNeely and Falci, 2004; Rawatlal and Petersen, 2012; Govender et al., 2013; Ungar and Theron, 2019; García-Moya, 2020). Teachers and educators are in a position to be able to provide support to AGYW, promote healthy SRH decision-making, and thereby help to decrease negative outcomes such as teenage pregnancy (Herrero Romero et al., 2019). Teacher support, defined as social, practical, or emotional support from a teacher, has been found to be independently associated with reduced HIV-risk behaviour incidence amongst adolescents in South Africa, with significant HIV prevention effects, independently of other social interventions (Cluver et al., 2016). For these reasons, adolescents’ sense of connectedness in the school setting has implications not only for academic success, but also for SRH outcomes such as teenage pregnancy (Thompson et al., 2006; Govender et al., 2013; Sharp et al., 2019).

The bulk of research examining school and teacher connectedness relates to the Global North and to high-income contexts. Additionally, few studies have differentiated between school connectedness and teacher connectedness (Garciìa-Moya et al., 2019). In light of this, and that the fact that school and teacher connectedness are likely to have socio-cultural and contextually specific dimensions, there has been a call for more comprehensive examination of teacher connectedness in different settings (Garciìa-Moya et al., 2019). There is a dearth of literature pertaining to student-teacher relationships and connectedness in the South African context, and in particular how these impact on mental health and wellbeing, and SRH amongst AGYW. A better understanding of the intersecting social cohesion processes as a protective factor against adolescent health risk behaviours is necessary (Govender et al., 2013). The aim of this study was to explore barriers and facilitators to school attendance, retention, and attainment amongst AGYW in communities in South Africa characterised by high rates of teenage pregnancy and HIV. Additionally, this study sought to provide evidence to improve the provision of necessary support to both AGYW and teachers, in order to strengthen AGYW educational motivation, aspirations and achievement. In this paper, we focus on student-teacher relationships and their impact on the educational attainment, mental health and well-being of AGYW in South Africa.

## Materials and methods

### Study setting

Data collection took place between August 2018 and March 2019 in five districts across five provinces of South Africa: City of Cape Town, Western Cape (WC); King Cetshwayo, KwaZuluNatal (KZN); Gert Sibande, Mpumalanga (MPU); Bojanala, North West (NW); and Nelson Mandela Bay, Eastern Cape (EC). Selected districts were a mix of urban, semi-urban and rural, but shared the characteristic of having amongst the highest teenage pregnancy and HIV incidence rates nationwide.

### Sample

The study sample consisted of a total of 237 adolescent girls and young women (AGYW) aged between 15 and 24 years. Of these, 177 were in the 15–19 years age group, and 60 were in the 20–24 years age group. Additionally, the sample included 10 school teachers. Participants were purposively sampled from selected schools within each study district. The sample of teachers included those who were involved in teaching Life Orientation^1^ and/or involved with non-academic extracurricular activities, and/or those that had been liaising with coordinators in a school-based health intervention.

### Data collection

Data collection comprised of 63 in-depth interviews (IDIs) and 24 focus group discussions (FGDs) with 237 AGYW, and IDIs with the 10 teachers. Trained female researchers fluent in the local languages conducted IDIs (20–40 min in length) and FGDs (40–90 min in length) in participants’ language of choice (English, isiZulu, isiXhosa, Setswana, or siSwati). Interviews and FGDs were semi-structured, following topic guides with open-ended questions and probes for potential additional issues, allowing for iteration, probing and digression on relevant themes. School connectedness and teacher connectedness were not initially a focal area in this study, but emerged as salient topics during data collection. These themes emerged in response to questions in the topic guides relating to perceived barriers and facilitators to school attendance, retention, and achievement and perceptions of received and needed support. AGYW were asked to describe their overall school experience, and the extent to which they received support from various people in their lives, including teachers. Teachers were asked questions relating to their perceptions of the support received and needed by AGYW at school, and outside of school. In this paper we focus on findings related to student-teacher relationships. A brief demographic questionnaire was also administered to participants.

### Data analysis

Translation followed a four-step approach. Audio recordings of IDIs and FGDs underwent a process in which they were: (1) transcribed verbatim into their original language; (2) reviewed by the interviewer/s for accuracy; (3) translated into English; (4) and reviewed again to assess the accuracy of translations. Following a cyclical thematic approach, analysis started with pre-determined deductive codes, based on the discussion topics, which underwent inductive development and refinement as the analysis progressed (Bradley et al., 2007; Vaismoradi et al., 2016; Nowell et al., 2017). Collaborative interpretation by research team members involved data immersion and familiarisation, and repeated transcript readings through which meanings and patterns emerged. Theoretical and reflective thoughts that developed through immersion in the data were shared amongst the research team. Codes were identified, refined, and entered into NVivo 12 software, which was used to enable the organisation and labelling of relevant text from the raw data. As concepts and themes inductively emerged, they were collaboratively reviewed and refined. Weekly meetings, held throughout the data collection and analysis phases, allowed for team debriefing and discussion of the evolving connection and interpretation of the data. To accompany the coding process, and ensure reliability and validity of the analysts’ interpretations, participant verification feedback workshops were held with AGYW. A total of three workshops were held with 32 AGYW aged 15–24 at three of the study sites, some of whom had previously participated in IDIs and FGDs, and some who had not. Workshop participants’ feedback, captured through notes and transcribed audio recordings, are presented alongside the findings.

### Ethical considerations

Informed consent was obtained from all participants 18 years and older. Written assent with written guardian consent was obtained for those younger than 18 years. Reimbursement and refreshments were provided. The study protocol and research tools were approved by the South African Medical Research Council Research Ethics Committee, and the interviewers were trained in human subject research ethics.

## Findings

### Demographic characteristics

Amongst the 237 AGYW respondents aged 15–24, the mean age was 17.4 years. Self-reporting on the highest level of education achieved amongst the 237 AGYW, 2% (*n* = 5) some primary schooling, 83% (*n* = 197) were either currently in school, or had achieved some secondary schooling, and 11% (*n* = 25) had completed secondary schooling. A total of 3% (*n* = 7) had some tertiary education (college or university). Out of all AGYW aged 15–24, 18% (*n* = 41) self-reported to have had ever been pregnant. The ten teacher respondents, three male and seven female, were aged between 27 and 57 years, with a mean age of 45 years.

The findings presented below are arranged into key thematic areas that emerged during analysis relating to AGYW’s narratives of their relationships with teachers. Quotations are English translations, followed by details of the participant’s province, sample group.

### Adolescent girls and young women narratives

When asked to describe their overall school experience, the qualitative narratives shared by AGYW demonstrated the disconnect they felt in relationships with their teachers, characterised by an overwhelming sense of mistrust: “We don’t trust our teachers” (WC, 15–19 years). In addition to lacking trust, AGYW described feeling “scared that they (teachers) are going to judge us” (NW, 15–19 years). The fear of judgement fosters a reluctance amongst AGYW to confide in or seek support from teachers: “Girls here are secretive because the teachers here judge” (WC, 15–19 years). In addition to fearing judgement, mistrust of teachers related to breaches of confidentiality: “Teachers do not always maintain confidentiality of the issues we share with them. You’ll be surprised how information you shared with one teacher got to be known by another teacher” (WC, 15–19 years). Emerging clearly in the data was the perception amongst AGYW that teachers gossip with each other about learners: “Teachers, when they are at the staff room... they talk about you... their topic will be you” (WC, 15–19 years); “They’ll talk about us in the staff room... whatever you’re going to talk to her (teacher) about won’t stay with her for long... they’ll go around telling other teachers” (NW, 15–19 years); “Teachers sometimes gossip and tell other teachers at the staff room about things that you told them in confidence” (MPU, 15–19 years). Those AGYW who trust certain teachers enough to confide in them, risk having their confidentiality breached: “In this school we have teachers that you can go to share some things, deep things, but the problem starts when the teachers gossip to other teachers” (WC, 15–19 years).

The lack of trust AGYW have in teachers, leads them to avoid seeking support: “They (teachers) have that weakness (gossiping)... that’s why we are now afraid to tell them our problem” (MPU, 20–24 years). AGYW suggested that consulting or confiding in a teacher about sexual health would likely result in private information being shared with other staff members, and even with other learners. “There are teachers here at school who talk about, like you would tell a teacher that you are on contraceptives... that teacher will discuss your problem with another teacher, and that teacher will tell another one. You start to feel uncomfortable... sometimes they make examples about you... in the LO (Life Orientation) class” (WC, 15–19 years). The perceived inability of teachers to maintain confidentiality was viewed by AGYW as a sign of disrespect toward learners: “They (teachers) don’t have respect for us... we have deep secrets... we go to a specific teacher that we trust and now that teacher will go to another teacher to go gossip about us... that teacher maybe has learners around her desk and she will tell her learners and those learners will tell others now the whole school looks at you” (WC, 15–19 years).

Not being able to trust teachers or seek their support means that AGYW feel they have no one to confide in, making them feel emotionally isolated: “You cannot trust anyone... teachers here at school like to gossip” (MPU, 20–24 years). This sense of emotional isolation and lack of support negatively impacts the mental health and well-being of AGYW: “We can’t share things with our teachers. They are supposed to be our parents at school, but we can’t share things with them, because they can’t keep it with them... they will share it with other people, then you will see people giving you funny looks, so we don’t share our sexual and personal life things with them, because of how they are. (Instead) we keep it to ourselves, then some of us commit suicide” (WC, 15–19 years). The perception amongst AGYW that teachers do not care about their feelings or problems was described as hurtful: “There are those teachers when you tell them your challenge, they ignore you... That hurts school learners” (MPU, 20–24 years).

Instead of feeling that teachers provide support and encouragement, AGYW described teachers enacting behaviours that negatively impact learners: “We have those kinds of teachers who instead of encouraging you, will discourage you and keep pushing you down” (KZN, 15–19 years). Disparaging remarks from teachers compound the sense of disconnect: “Our teacher will tell you that you are rubbish... how are you going to talk to the teacher if you are a rubbish?” (WC, 15–19 years). Name-calling or ridicule from teachers negatively affect the self-esteem and self-confidence of learners: “Our teacher calls me by those names in front of a full class and you feel small. He will call me stupid... all sorts of names. That makes you feel small” (MPU, 15–19 years). Discouraging attitudes displayed by teachers negatively impacts on AGYW self-confidence, motivation, as well as academic performance: “When the teacher has an attitude against you, you end up doing worse than you were doing before” (WC, 15–19 years).

The motivation and willingness of AGYW to attend school is also negatively affected by discouraging and negative attitudes expressed by teachers: “it will make you feel like not going to school like when a teacher will say to you “this thing that keeps on failing”... you end up hating to go to school, and your self-esteem becomes down” (KZN, 15–19 years); “Teachers will be like “...you should have stayed at home and not come to school”... (so) the following morning you will be like why should I go to school? The teacher said I might as well stay at home” (WC, 15–19 years). The sense of being disrespected or ridiculed by teachers negatively impacts on school attendance, particularly for learners who are more vulnerable or require additional support: “Some children do not come to school because of teachers, because of teachers’ attitude. They don’t treat other kids well... They disrespect them because they are not clever... they make a joke of them. These kids then end up losing that love for school... then drop out of school because they are scared to be laughed at when they have failed” (MPU, feedback workshop).

The sentiment was also expressed by AGYW that teachers lack passion in their work, and as a consequence, fail to inspire learners: “They don’t have passion... they will be like “I don’t care about you, my money (salary) is in”... they don’t inspire us” (WC, 15–19 years). Respondents described the way in which the quality of instruction and education provided is negatively affected by factors such as teachers being distracted by their mobile phones, or giving rushed and incomplete classes: “teachers become bored... they spend most of their time on their phones instead of teaching. And when they teach, they are in a hurry and miss other points” (WC, 15–19 years).

When learners seek career advice or support, they are ridiculed, which causes feelings of embarrassment and consequential reluctance to seek the help and advice they need to pursue their career aspirations: “When you go and ask them what subjects you must do to become a lawyer, they will answer with rage and say at your age you don’t know what subjects you want to do? ...you will be disappointed with that answer because you were not expecting it and end up saying “it’s fine teacher thank you.” You end up lying saying you are rushing, it’s not that you are rushing somewhere but because you are embarrassed because of what the teacher said to you. And they will repeat that again in class tomorrow in front of people. You become embarrassed and not knowing what to do in life, because she’s not telling you what you should do to succeed” (WC, 15–19 years).

Adolescent girls and young women respondents described scenarios in which pregnant learners were mocked and ridiculed by teachers in the classroom: “In Life Orientation class it will seems when the teachers are talking (about pregnancy) they are referring to her (pregnant girl in class)... she will just remain quiet because she doesn’t want to be teased... the teacher will blame her saying “after all you pregnant.” (WC, 15–19). In addition to pregnancy learners being treated badly by teachers, AGYW described situations in which HIV positive learners were treated disrespectfully, with teachers discussing learners’ HIV status with others: “If it happens that a learner is diagnosed (HIV) positive... teachers shouldn’t talk about it because some people have fragile hearts and they get hurt easily. We wish they could keep things private... teachers should not expose our problems to other children and they make fun about it” (WC, 15–19 years).

Another factor adding to the sense of mistrust in teachers related to AGYW experiences of having been victimised or sexually harassed by male teachers, and when reporting this harassment they are not taken seriously: “Male teachers... they will create a story about you even when you did not do that particular thing. I was once a victimised by a teacher... he just said I wanted him to be my partner... That made me feel offended, so we went to him with my friends and confronted him about it. The sad part was that he said all this in front of other teachers... his response was that he was just joking with me... I then came to school and spoke to one lady teacher, told her that I’m hurt by what the teacher has said about me... I was so angry, she then said I must calm down” (MPU, 15–19 years). Respondents suggested that male teachers sometimes make inappropriate comments of a sexual nature to female learners: “One male teacher... he would say “look me straight into the eye not in front of my trouser” (at his crotch), disturbing the learners... that does not sit well with me” (MPU, 15–19 years). Allegations of teachers’ coercive sexual harassment of female learners emerged in the narratives of AGYW respondents: “Teachers at school are making advances on the school girls and if they don’t agree, they abuse them” (MPU, 20–24 years).

In general, AGYW respondents voiced their desire for improved, non-judgemental, holistic support from teachers: “We would like not to be judged by our teachers... they must not humiliate you in class when you do something wrong in front of other children” (WC, 15–19 years). The desire for improved confidentiality, and more respectful communication, were amongst the key demands AGYW respondents expressed: “(We want) privacy, teachers should not expose our problems to other children and make fun about it” (WC, 15–19 years); “When you go to (a teacher) in confidence with some confidential issue. Maybe you told him something that has happened at home, we request that they must not share the information we have shared in confidence with them, with their colleagues” (MPU, 20–24 years). Respondents suggested that if teachers were more supportive, there would be beneficial effects on levels of motivation amongst learners: “If teachers could be supportive and friendly... maybe we can show more interest in our studies” (WC, 15–19 years).

The view was expressed that teachers are in a position to act as positive role models for learners, setting examples of respectful and pro-social behaviour: “teachers must be taught to respect learners, so that they won’t spread rumours about school children, so that they can set an example for us as school children, because they fail to respect school children. That will also cause us not to respect fellow learners. So, respect from teachers to learners would be a good idea, that will encourage students to respect each other and there won’t be people who gossip, laugh at others and do all those things” (EC, 15–19 years). When parents are not available or present, or in situations where AGYW do not reside with parents, teachers could play the role of a supportive adult/caregiver: “Our parents are staying very far, we take them (teachers) as our parents. So, if I tell them my problem in confidence and they tell somebody else, etc., then it is no longer a secret” (MPU, 20–24 years). In our analysis of the data, there were very few instances in which AGYW shared their positive experiences of having received support from teacher and educators. There was recognition of the capacity for educators to play a positive parental role: “The teachers and the Principal at the school are like our parents because they play a role in our lives... (they) show us positive things in our lives” (WC, 15–19 years). Importantly, it was noted by AGYW themselves that not all teachers are the same: “there are those kind and approachable teachers whom you can divulge any kind of challenge you are facing. There are those when you tell them your challenge, they ignore you” (MPU, 20–24 years).

Despite the majority of AGYW expressing mistrust in teachers, it was highlighted that not all teachers are the same, and there are a rare few who can be trusted: “There are those kind and approachable teachers whom you can divulge any kind of challenge you are facing” (MPU, 20–24 years).

### Teachers’ narratives

Those teachers interviewed shared their views on the roles and responsibilities that teachers have toward AGYW learners. Respondents felt that teachers should provide psychosocial support, emotional support, and care for the well-being of AGYW: “You see what the girl needs, the girls first need love... Just love... from home and at school, you should bring the girl child closer to you so you can see all the changes that will happen to her... If you do so, then you will give her all the support, because you won’t be able to support her if she’s away from you... when you bring them closer, that’s where you’ll see how much support she needs, but if you are a person who doesn’t pay attention, you will not see that she needs support... Maybe you just notice, if someone is absent you notice that she has been absent so many times, and call her aside, what happened, what’s causing you to not come to school, then you find out that there is a problem like that and that. And also boys, same thing... But girls, it’s necessary to be closer to them” (KZN, teacher). Building the self-confidence and self-esteem of AGYW was seen as of critical importance: “The biggest thing that we (teachers) are supposed to do, we are supposed to build that spirit of confidence in children right, I mean a child who doesn’t have confidence in themselves... it will build up to a point where it causes her harm... if we can teach our children to talk... and they should talk... in life they should know who to confide in, people they trust, irrespective of whether it’s a parent at home or a teacher” (NW, teacher).

Understanding the emotional and psychosocial vulnerability of AGYW was described as important in order to be able to support them appropriately: “For girls to feel loved, we shouldn’t criticise them even when they have done wrong, we can say things that go along with the situation, but we need to get to the bottom of all the reasons that have led them to behave in that manner, where did it start, because sometimes leaners find themselves in situations because they lack support at home, and when the learner arrives at school, other learners abuse them, though it might not be known, then they end up misbehaving because they have pressure somewhere” (KZN, teacher). It was suggested that AGYW are not always able to seek emotional support or communicate effectively about their needs: “The problem we have is that our learners are not used to voicing out things that are affecting them personally... Some are not used to that... They know that they won’t ask... just anyone, who will ask a lot of questions... they will look for a person that they trust and someone who will be understanding of the situation they are facing” (KZN, teacher).

Helping AGYW cope with stress and anxiety related to exams and academic performance was also described as within teachers’ responsibilities: “Mental health support, eh yes, I used to give them sometimes, because in some learners, problems will start now that exams will commence, there will be a lot of problems that will arise maybe some leaners experience fear... some are scared of failing... it’s their expectations, they think that even though they have studied, they might not remember when it is time to write, so we support them spiritually and physically for the exam... You will find others will be so tense that they don’t even give themselves time to eat” (KZN, teacher).

Teachers felt they had a responsibility to play parenting roles toward AGYW, in addition to playing the role of educators: “It’s not that here at school... it’s just teaching and learning, and it is all over... the class teacher should serve as a parent” (KZN, teacher). Additional burden is placed on teachers where parental involvement is lacking or insufficient: “(Female learners) need support, they need guidance, and one key challenge that we have in our school community, and one key challenge we have in our school, in our community, is no parental involvement. Parental involvement is a big challenge... our learners come from a very poor background, where social ills are the order of the day. So the parent doesn’t care... doesn’t care whatever the child does... the difference is... the school stands in for the parents” (EC, teacher). Respondents described the way in which their maternal/parental responsibilities toward learners, feeling responsible for their holistic well-being, causes additional stress to already overburdened teachers: “I end up being a mother to a lot of children, some are boys, some are girls, so, sometimes it becomes a challenge because I reach home exhausted... You will find that I was talking for the whole day and it exhausts me... there’s a trend that is going on at school that I want to put to an end because I realised that it was becoming too much, let’s say there’s a learner who has a problem at school, even if I’m in class I will be called, they will refer that learner to me whereas we are all allocated a class, I also have learners in my class... it becomes a problem if a teacher cannot deal with a minor issue, I also realised that it was too much for me. ...rather they come if it is a major problem, a major problem, not one they can deal with, so I’ve seen that that challenges me a lot but I was helping no matter how tired I was... it was becoming too much because I saw an element of being irresponsible from other colleagues” (KZN, teacher).

Teachers described the ways in which they try to help and support learners, particularly those who come from socio-economically disadvantaged backgrounds: “At our school our children is our gold, so anything we do at this school, we do it with a passion... what we do, we look at children, we identify... underprivileged children, children with needs, whatever the case may be... we look at... where we can provide them with school clothes... where we can give personal counselling... they must be free to come... and share whatever they encounter” (EC, teacher). Teachers who have vulnerable learners in their classes end up getting financially and emotionally involved in situations where learners are faced with poverty, abuse or neglect: “I have a challenge of this learner who I tried to help... (she) does not have a place to stay... and she was emotionally abused... (her mother) doesn’t support her... she gets the grant, her mom receives the money but she doesn’t support her with anything. As far as transport, she has to make her plan to see how she will come to school and how she will get home... they almost destroyed all her uniform, she was only left with a shirt and jersey... So it’s still a challenge... I took it up with the (school) management thinking that they will call the parent and find out what’s happening, but because of work and lack of time, nothing has happened thus far, but I thank God that up to this far, the learner still comes to school because this thing started in the beginning of the year, so seeing her still coming, it’s a relief... Though sometimes she comes to me and says she doesn’t even have food, she doesn’t have this and that, but because I have pads here at school, I provide her with pads, but the uniform issue is still outstanding... she doesn’t have uniform ...I see this child ending up being exposed to different things... life is challenging her in all aspects, and she is a girl... if I did not give her money... and I pack food for her to take home... That is the challenge I am faced with now” (KZN, teacher).

Teachers often feel overwhelmed when they are left to deal with serious situations in which AGYW learners have been abused at home: “We have cases that are sometimes brought to us here at school by neighbours... some neighbours are very attentive, they can see... or a child is the one who tells the neighbours whatever happens in their house... they end up being brought by the neighbours at school... It becomes too much for us because we are here to teach the learner, we end up with cases which requires the police, and we wonder why they brought those things to us” (NW, teacher). At times abused learners seek assistance themselves: “When a child comes honestly, confessing that he/she needs help, we attend such child... some are beaten up... beaten too much at home that they end up with bruises... they come with cases which are serious sometimes” (NW, teacher).

Some teachers highlighted that although they would like to be able to provide learners with psychosocial and emotional support, they don’t have the time or capacity to do so sufficiently: “We don’t get time to deal deep with the issues” (MPU, teacher). Teachers also suggested that schools lack support structures for vulnerable learners, or those who engage in risk behaviours such as substance use.

## Discussion

Emerging clearly in our analysis of the data from IDIs and FGDs with AGYW was the sentiment of the lack of trust that AGYW have in their teachers. Mistrust in teachers’ ability to maintain confidentiality was cited as a major issue, with AGYW respondents sharing experiences of confidentiality being breached after confiding in teachers around sexual health issues. AGYW expressed sentiments of having their trust betrayed when teachers gossip about them. Although teachers are in a position to provide critical psychosocial and emotional support, AGYW feel unable and unwilling to confide in, and seek support from teachers, demonstrating a sense of student-teacher disconnect. The lack of effective communication and emotional support from teachers fosters a sense of isolation amongst AGYW, and negatively impacts their mental health, and school performance. AGYW respondents voiced a desire for improved communication with, and increased emotional support from teachers, suggesting that teachers would be appropriate adult support mechanisms. It is evident that some teachers themselves recognise that AGYW need psychosocial support, particularly those learners who are socio-economically disadvantaged. However, teachers often feel overwhelmed and lack the capacity to provide learners with the support they need. Overall, both AGYW and teacher narratives depicted a lack of teacher connectedness in these settings.

In our study, AGYW respondents described ways in which their lack of connectedness with teachers has negative impacts on their mental health and well-being. The feeling of not being able to trust teachers or seek their support means that AGYW feel they have no one to confide in, leading to feelings of emotional isolation. In addition, the harsh, ridiculing or judgemental words that some teachers direct at learners negatively affects their self-esteem and self-confidence. It is possible that the perspectives of learners and teachers may be somewhat discordant. AGYW’s subjective experiences of feeling judged or ridiculed, are likely to negatively impact their mental health, and could potentially have serious consequences. For example, a study in the Gauteng province of South Africa found that feelings of sadness, discouragement, worthlessness, suicidal ideation and loss of opportunities amongst learners was due to negative relationships with, and disrespectful treatment from teachers (Naicker et al., 2014). The level of connectedness between students and their teachers can have a strong impact on the learning experience, and the mental health of students (Gillespie, 2002). Evidence suggests that teacher connectedness may help to reduce rates of depression and suicidal ideation amongst students (Govender et al., 2013; Joyce and Early, 2014; Sharp et al., 2019). Where students feel that teachers care about their well-being, adolescents are less likely to experience depressive symptoms, and more likely to have future positive emotional well-being (Joyce and Early, 2014). Teacher connectedness has been framed as a psychological resilience factor for students in low-resource settings; and shown to be negatively correlated with emotional distress, suicidality, violence, and substance use in these contexts (Sharp et al., 2019). Caring and supportive relationships between teachers and adolescents can serve a protective function, acting as buffers from adverse effects or risk, particularly for students who are at risk socially and academically (Davis, 2006; Baker et al., 2008). The protective effect that having a personal connection with a teacher has on students’ educational, behavioural and health outcomes is amplified amongst low-income students, students who live in unsafe or violent communities, students who lack parental/familial support, and female adolescents (Joyce and Early, 2014; Lenzi et al., 2017; Duong et al., 2019; Garciìa-Moya et al., 2019). Students who are economically or socially vulnerable, and are at a disadvantage for educational attainment, can succeed and achieve academic success in the presence of significant obstacles, if they have strong relationships, characterised by respect, trust, care, with at least one teacher who serves as a positive role model and supportive and caring adult (Downey, 2008).

Adolescent girls and young women respondents in our study expressed consternation over the lack of respect that teachers have for them, evident in the way that teachers ridicule or mock students in class. Mutual respect is one critical component for building trustworthy and supportive student-teacher relationships. Key teacher behaviours that contribute to building close student-teacher bonds include holding frequent social conversations with students about their life outside of the classroom, increasing teacher accessibility and availability, and showing respect for students by valuing their perspectives and ideas (Liebenberg et al., 2015; Binfet and Passmore, 2017). In the narratives of AGYW in our study were descriptions of pregnant learners being mocked and judged by teachers, particularly in Life Orientation classes. In order to avoid negative consequences on AGYW educational and future opportunities, it is critical that teachers’ moralising or judgemental opinions toward learners who most need support, such as those who are pregnant or already have children, are addressed, and that teachers are responsive to the support needs of this sizeable, and vulnerable group (Bhana et al., 2010). Also relating to a lack of respect for learners, some AGYW in our study shared their experiences of being sexually harassed by or receiving inappropriate remarks from male teachers. There have been prior allegations of sexual abuse of AGYW by male teachers in South African schools (Bhana et al., 2010). It was outside the scope of this study to explore this issue in detail, but it warrants further investigation.

Most AGYW respondents described a reluctance to confide in teachers, due to the belief that teachers discuss learners’ confidential information with colleagues and in the staff room. It is possible that one factor that might explain AGYW feelings that teachers breach confidentiality when they confide in them may be due to teachers needing to refer learners for services in situations where they require additional professional help, or where a teacher is unable to assist alone and requires support from colleagues. The dimension of trust is a critical element in the interpersonal teacher-student relationship; it has been suggested that students generally expect teachers to be trustworthy, automatically trusting a teacher until the teacher violates that trust (Dobransky and Frymier, 2004). Trust is an inherent part of teacher connectedness, a foundation for open communication and information sharing, and key for the creation of an environment in which students feel affirmed and supported (Gillespie, 2002; Mitchell et al., 2016). Students who have strong bonds with, and trust, their teachers are more likely to seek their help and guidance, thereby accessing teachers as a key source of support (Baker et al., 2008; Anderson et al., 2011; Lenzi et al., 2017).

There is an important distinction between received and perceived social support; students’ perception of teacher support being a critical component of teacher connectedness (García-Moya, 2020). The perception of being listened to and cared for, and enacting “help-seeking” behaviour through identifying people or resources in order to solve a problem or address a concern, is a crucial component of coping behaviour, necessary for good mental health (Van Der Riet and Knoetze, 2016; Lenzi et al., 2017; Duby et al., 2021). Trust is also a critical aspect of the help-seeking process; when students trust their teachers, they are more likely to confide in them (Mitchell et al., 2016). A lack of trust, or the concern that personal disclosures may not be kept confidential serve as barriers to help-seeking; conversely, a high level of trust or an emphasis on confidentiality in a relationship, facilitate help-seeking (Van Der Riet and Knoetze, 2016). Interpersonal relationships characterised by social trust have significant positive impacts on the academic and psychological well-being of adolescents (Roffey, 2012). The feeling of having a reliable and trustworthy source of support is critical for good mental health. When students perceive their teachers as caring, trustworthy and supportive, they are much more likely to have positive academic and health outcomes (Davis, 2006; Baker et al., 2008; Duby et al., 2021).

Teacher respondents in our study shared their perceptions of the multiple roles that teachers should play in the school environment, expressing the view that their responsibilities toward learners go above and beyond academic instruction, and include the provision of support. Congruent with AGYW respondents’ narratives of the importance of teacher support for those students whose parents are absent or unavailable to provide sufficient support, teachers described their attempts to support vulnerable learners as much as possible, sharing how this responsibility places additional stress on them. Compounded by difficult working conditions in government schools, teachers described feeling overwhelmed with the multiple roles and responsibilities they have to fulfil, in order to provide the psychosocial support that AGYW need in addition to academic support. This was exacerbated in situations where teachers identified learners in their class who were facing circumstances of poverty, abuse, or neglect. As our teacher respondents suggested, at times they become caught up in the well-being of students, especially those who face challenges related to poverty or violence. The emotional strain and time burden that is placed on teachers who are increasingly expected to foster students’ social and emotional competencies alongside the development of students’ intellectual development and corresponding academic achievement, can be overwhelming (Binfet and Passmore, 2017). This increased expectation of teachers’ roles is partly due to a recognition that many learners, especially those in socio-economically difficult circumstances, come to school underprepared materially and emotionally for optimal functioning and learning (Binfet and Passmore, 2017). Teachers in the South African context bear an additional burden due to the disruption of families caused by a combination of social, economic and historic factors such as migrant labour and apartheid policies. Indeed, figures published in 2017 suggest that 21% of South African children do not live with either of their biological parents (Sharp et al., 2019).

The provision of psycho-social and emotional support to students is ideally part of the teaching package, with teachers serving as a primary social and emotional support mechanism for some students (Binfet and Passmore, 2017). Teachers often have to be mother/parent, psychologist/counsellor, friend, spiritual advisor, as well as academic educator; as described by teachers in our study, playing these multiple roles can be challenging (Hattingh and de Kock, 2008). Even if educational policies outline the concept of an ideal teacher, the reality of resource and material constraints can impede the achievement of this ideal (Harley et al., 2000). For teachers working in under-resourced schools, with oversubscribed classes, the roles of counselling and pastoral care to students may not be prioritised (Harley et al., 2000). A key aspect determining student-teacher relationships is the way in which teachers regulate and express their own negative emotions, which is often challenging given the stressful situations they have to deal with in the school setting (Jennings and Greenberg, 2009). Even when teachers recognise the importance of building positive relationships with students, if teachers feel overwhelmed or stressed, they are less likely to exhibit a caring attitude, provide support, or make an effort to foster connectedness with students (Jennings and Greenberg, 2009; Duong et al., 2019). The delivery of quality teaching and education in the South African state education system is problematic, due to factors such as shortages of teaching staff, low morale and poor working conditions (Fourie and Deacon, 2015). In circumstances such as these, it is unlikely that teachers are able to provide the sufficient academic support to learners, let alone the kinds of psychosocial and emotional support that would help to foster teacher connectedness. It is unfair to lay the blame upon teachers for the student-teacher disconnect, as teachers in the South African state system lack the necessary support, training and capacity to enable them to build relationships of trust with learners (Salmon and Sayed, 2016).

The main limitation of our study relates to the selection bias in the small sample of teachers interviewed. Sampled teachers included those who were already engaged in extracurricular activities at the schools, and/or teaching Life Orientation classes. Therefore, in addition to the likelihood that our sample included only those teachers who were more committed and engaged, and therefore more likely to be supportive of AGYW, it is also possible that as a consequence of social desirability bias, teachers would also be unlikely to admit their own lack of support of learners. As school connectedness was not an initial focus of the study, interview guides did not include specific questions on teachers’ connectedness or relationships with leaners, but rather this data emerged in discussions centred around support that AGYW receive or need in the school environment.

### Implications for practice

Interventions and programmes that can help to foster a sense of school connectedness and teacher connectedness amongst students are critical in order to harness the potential of schools as a context through which to provide necessary psychosocial support, and promote the well-being and mental health of young people in South Africa. Efforts to facilitate more effective support for AGYW in their SRH decision-making and behaviour, need to include the provision of integrated health delivery in schools, of which mental health promotion is a key component.

Support from teachers can be an effective form of social support which helps to reduce risk behaviours amongst adolescents; tailored combination social protection, inclusive of teacher support, is likely to be the most effective approach to reducing HIV risk amongst this age group (Cluver et al., 2016). The ability of South African teachers to engage and connect with their students may be improved by addressing poor working conditions (Fourie and Deacon, 2015). Teachers need to be supported in order to experience more meaning in their work, which would then enable them to make a positive difference in learners’ lives through building positive, trusting relationships (Fourie and Deacon, 2015). Additionally, special consideration needs to be given toward providing teachers with professional development in order to equip them with the skills with which to provide support to pregnant learners, teenage parents, and those learners living with HIV (Bhana et al., 2010).

Teachers can play a critical role in promoting school connectedness through positive relationships with learners, providing mentorship, role modelling healthy behaviours, and building a positive school climate conducive to learning and a culture of well-being (Rawatlal and Petersen, 2012; Kern et al., 2017). In order to achieve this, efforts need to be made to build teachers’ interpersonal skills and competence, enabling them to foster positive relationships with students (Rowe et al., 2007; Joyce and Early, 2014). Teacher training should help to enrich teachers’ understanding of how levels of school connectedness and the school climate can influence learners’ academic achievement, positive peer interactions, social acceptance, and overall emotional well-being, particularly in contexts where young people may lack positive parental role models or familial support (Rawatlal and Petersen, 2012; Kern et al., 2017).

The potential to use schools as conduits for promoting adolescent mental health and providing linkages to mental healthcare are increasingly being recognised, with school staff being in a unique position to support positive psychosocial outcomes amongst vulnerable young people (Kutcher and Wei, 2012; Liebenberg et al., 2015; Kutcher et al., 2016). School-based mental health interventions should include a broad spectrum of prevention, referral, assessment, intervention, and counselling. Evidence based interventions to address school connectedness and promote mental health amongst students include the provision of training and enhanced staff education to improve the mental health literacy of teachers and equip them with the skills to identify common presentations of mental health issues, and pick up on early warning signs indicative of stress, anxiety, trauma, abuse, depression (Kutcher et al., 2016; Kern et al., 2017). Standard teacher training and professional development programmes need to include components on social and emotional developmental processes during childhood and adolescence, and incorporate curricula targetted at the most common mental health issues likely to be present in schools, and those which may affect school attendance and performance (Jennings and Greenberg, 2009). Training teachers on how to identify a learner who is showing signs of disengagement and disconnectedness, and how and when to refer them for psychosocial support would also help to increase connectedness (Kern et al., 2017). The capacity of teachers to connect their learners to appropriate mental health support should also be enhanced, alongside instruction on how best to approach learners in a way that encourages them to discuss their concerns and feelings (Kern et al., 2017). Equipping teachers with skills to assist in the identification and referral of mental health issues amongst learners may also help to address their own sense of feeling overwhelmed by the emotional and behavioural challenges in their classrooms (Fazel et al., 2014).

Interventions and programmes that can enable increased student–teacher communication and bonding, and foster caring relationships in the school setting, could provide an important psychosocial support mechanism for young people, and promote positive emotional, social and educational development (Chapman et al., 2013). Providing teachers with skills training on how to respond to students using strategies such as supportive listening and praise, would help to improve the quality of student-teacher relationships (Kincade et al., 2020). However, training teachers on these practices may not lead to sustained implementation alone; there is a need for on-going training and consultation to ensure teacher buy-in, and support teachers’ adoption, delivery, and sustained use of these practices (Kincade et al., 2020). Interventions that have shown success in improving student-teacher relationships and building students’ trust in teachers include mentorship or internship programmes and relationship-focused reflection (Joyce and Early, 2014; Lenzi et al., 2017).

## Conclusion

Our findings provide valuable new insights into student-teacher relationships in the South African context, how the student-teacher disconnect experienced by AGYW impacts not only on AGYW’s educational attainment, but also on their mental health and well-being, as well as their sexual and reproductive health. As is evident from our findings, AGYW desire and need better support from teachers. It is likely that more trusting and supportive relationships between AGYW and teachers would improve the potential of AGYW for educational attainment and help to decrease rates of teenage pregnancy. Addressing the disconnect between AGYW and their teachers may go some way to improving AGYW’s perceived psychosocial and emotional support, and in turn, lead to reduced engagement in risk behaviours, mitigating their risk of negative sexual and reproductive health outcomes, and reducing the prevalence of teenage pregnancy and HIV amongst AGYW in South Africa.

## Data Availability

The raw data supporting the conclusions of this article will be made available by the authors, without undue reservation.

## Footnotes

1 The Life Orientation curriculum in South Africa includes aspects related to adolescents’ sexual and reproductive health. For more information visit: https://www.education.gov.za/.
